# Selection of Vegetable Oils and Frying Cycles Influencing Acrylamide Formation in the Intermittently Fried Beef Nuggets

**DOI:** 10.3390/foods10020257

**Published:** 2021-01-27

**Authors:** Siti Nur Syahirah Ahmad, Azmil Haizam Ahmad Tarmizi, Raznim Arni Abd Razak, Selamat Jinap, Saparin Norliza, Rabiha Sulaiman, Maimunah Sanny

**Affiliations:** 1 Department of Food Science, Faculty of Food Science and Technology, Universiti Putra Malaysia (UPM), Serdang 43400, Selangor, Malaysia; syera1326@gmail.com (S.N.S.A.); sjinap@gmail.com (S.J.); 2 Product Development and Advisory Services Division, Malaysian Palm Oil Board, 6, Persiaran Institusi, Bandar Baru Bangi, Kajang 43000, Selangor, Malaysia; raznim@mpob.gov.my; 3 Laboratory of Food Safety and Food Integrity, Institute of Tropical Agriculture and Food Security, Universiti Putra Malaysia (UPM), Serdang 43400, Selangor, Malaysia; 4 Sime Darby Research Sdn. Bhd., Lot 2664, Jalan Pulau Carey, Pulau Carey 42960, Selangor, Malaysia; norliza.saparin@simedarbyplantation.com; 5 Department of Food Technology, Faculty of Food Science and Technology, Universiti Putra Malaysia (UPM), Serdang 43400, Selangor, Malaysia; rabiha@upm.edu.my; 6 Halal Products Research Institute, Universiti Putra Malaysia (UPM), Serdang 43400, Selangor, Malaysia

**Keywords:** acrylamide, intermittent frying, beef nugget, frying cycles, vegetable oils

## Abstract

This study aims to investigate the effect of different vegetable oils and frying cycles on acrylamide formation during the intermittent frying of beef nuggets. Different vegetable oils, palm olein (PO), red palm olein (RPO), sunflower oil (SFO), and soybean oil (SBO), were used for a total of 80 frying cycles. Oil was collected at every 16th frying cycle and analyzed for peroxide value (PV), *p*-anisidine value (*p*-AV), free fatty acid (FFA), total polar compound (TPC), polar compound fractions, and fatty acid composition (FAC). Total oxidation (TOTOX) value was calculated, and acrylamide content was quantified in the nuggets. Regardless of the oil type, PV, *p*-AV, and TOTOX initially increased but gradually decreased. However, FFA and TPC continued to develop across the 80 frying cycles. The C18:2/C16:0 remained almost unchanged in PO and RPO but dropped progressively in SFO and SBO. The lowest acrylamide content in fried products was observed in the PO, while the highest content was observed in RPO. Bivariate correlation analysis showed no significant (*p* ≤ 0.05) correlation between oil quality attributes and acrylamide concentration. The oil type but not the frying cycle significantly affected the acrylamide concentration in beef nuggets.

## 1. Introduction

Frying is one of the established cooking methods evidenced by high fried food consumption [1]. During frying, oil and food substances are exposed to moisture and oxygen at high temperatures, resulting in significant chemical reactions, namely the oxidation, hydrolysis, and polymerization. Furthermore, these reactions led to the accumulation of lipid degradation compounds [2] and acrylamide [3] that are harmful to human health. In 2002, acrylamide was detected in heated starch-rich food, which was first reported by the Swedish National Food Administration and the University of Stockholm [4]. Acrylamide is categorized under Group 2A—probably carcinogenic to humans by the International Agency for Research on Cancer [5]. The European Food Safety Authority released its scientific opinion on acrylamide in food and concluded that based on evidence from animal studies, dietary exposure to acrylamide potentially increases the risk of developing cancer for consumers in all age groups [6]. Acrylamide is mainly formed because of the Maillard reaction via condensation of the amino group consisting of asparagine and carbonyl group of the reducing sugars during heat treatment at temperatures above 120 °C [7]. Many researchers have reported that lipid oxidation promotes acrylamide formation [8,9,10]. In lipid oxidation, acrylamide may result from its precursor, acrolein formed from glycerol dehydration and subsequent oxidation to develop acrylic acid, and thus acrylamide in the presence of asparagine [11,12,13]. 

Vegetable oils have been consumed as a source of essential fatty acids and energy for the human diet [14]. Vegetable oils such as palm oil, sunflower oil, soybean oil usually is in the form of a variety of products, i.e., fried foods [15], containing a different number of tocopherols and tocotrienols, which are known as fat-soluble-antioxidants [16]. Red palm oil is a red colored vegetable oil containing abundant content of fat-soluble antioxidants which are a mixture of carotene, tocopherols and tocotrienols [17,18,19]. Deep-fat frying changes the quality of the oil by hydrolysis, oxidation, and polymerization [20,21]. Previously, various studies have reported the type of oil affecting acrylamide formation in food samples [12,22,23]. In particular, Gökmen [24] suggested that both lipid unsaturation level and oxidation rate influence the amount of acrylamide content. Chemically, oils with a lower level of unsaturated triacylglycerols are primarily more stable for frying [25]. On the other hand, different researchers have shown that increasing frying cycles would increase the formation of acrylamide in French fries [26] and sweet potato [22]. However, other research works have demonstrated that frying cycles have no negative impact on acrylamide formation [12,27,28]. To date, the available literature gave inconclusive findings on the effect of oil type and frying cycle on acrylamide formation. Furthermore, the influences of oil quality attributes due to hydrolysis, oxidation, and polymerization with the increase in frying cycles on acrylamide formation were not systematically studied. As such, this issue is worth further investigation. 

Nuggets is one of the common examples of a widely consumed fried product and abundantly produced by the food industry [29]. Previous studies on this food segment, particularly on chicken nuggets, mainly focused on the quality of the nuggets and oil, without determining the acrylamide concentration in the nuggets and/or its correlation with lipid oxidation [30,31,32]. Like any other fried products, nuggets are also subjected to frying at high temperature, and therefore, the formation of acrylamide is of great concern. Soncu and Kolsarici [33] reported that the average acrylamide content in chicken nuggets was 71.42 ng/g. In 2016, the Food and Drug Administration [34] reported that the minimum acrylamide that could be detected in chicken nuggets is 19 ng/g. Previously, Gholami et al. [35] established 61.5 ng/g of acrylamide content in fried beef, whereas Ghasemian, Rezaei, Abedini, Poorazarang, and Ghaziani [3] recorded 12 ng/g as the highest acrylamide level detected in a fried beef burger patty. However, to the best of our knowledge, no data on the acrylamide levels in beef nuggets have been published. Thus, this study aims to investigate the effect of different vegetable oils and frying cycles on the acrylamide formation during the intermittent frying of beef nuggets.

## 2. Materials and Methods 

### 2.1. Sample Preparation

This study was performed using frozen partially fried beef nuggets purchased from a local supplier, i.e., Ramly Food Processing Sdn Bhd, Bangi, Selangor, Malaysia. Palm olein (PO), sunflower oil (SFO), and soybean oil (SBO) were bought from a supermarket in Putrajaya, Malaysia. Industrial grade red palm olein (RPO) was collected from a local palm oil refinery, and it was used as it is without blending with other oils. This industrial grade RPO is not available in the market as the Carotino RPO commercially in the market is a blend of 80% canola oil and 20% red palm oil [36]. The beef nuggets were stored at −18 °C and thawed for a minimum of two hours at room temperature before frying. The vegetable oils were kept at room temperature (25 °C) for less than a week before use. Beef nuggets and vegetable oils from the same lot numbers were used.

### 2.2. Frying Experiment

The frying experiment was performed in duplicates using two units of stainless steel fryer with the capacity of 5-L, according to Ahmad Tarmizi et al. [36] with minor modifications. Before frying, fresh oil was poured into a frying pot and then heated to 180 °C for 30 min. For each pot, a batch consisting of 150 g of beef nuggets were fried at 180 °C ± 5 °C for 3.5 min. The beef nuggets were processed for 3.5 min in every 30 min for 8 h a day for five consecutive days

After the 1st, 16th, 32nd, 48th, 64th, and 80th frying cycles, the beef nuggets were collected and dried on a paper towel, cooled to room temperature, crushed, and homogenized using a blender. All homogenized samples were labelled and kept in a zip-lock polyethylene bag at −20 °C for subsequent acrylamide analyses. At the end of each day of frying operation (i.e., after 16th, 32nd, 48th, 64th, and 80th frying cycles), about 0.45 L of oil was collected using two 0.25 L dark amber bottles. Nitrogen was then flushed into the samples and kept at −20 °C for physico-chemical analyses. Triplicate analysis of free fatty acid, peroxide value, *p*-anisidine value, total polar compounds and polar compound fractions, and fatty acid composition were performed on the collected oil. Before commencing each day of the experiment, fresh oil was poured into frying pot to keep the oil at a constant level of 5 L.

### 2.3. Analysis Methods

#### 2.3.1. Chemicals and Reagents

Acrylamide (purity of >99%), trimethylamine, anisidine reagent, lipid standards, and phenolphthalein indicator were obtained from Sigma-Aldrich (St. Louis, MI, USA). Next, (^13^C3)-acrylamide (99% isotopic purity, 1 mg/mL) was purchased from Cambridge Isotope Laboratories, Inc. (Andover, MA, USA). Methanol, ethyl-acetate, hexane, hydrobromic acid (47% *w*/*v*), bromine (99.99%), potassium bromide, sodium sulphate anhydrous, sodium thiosulphate, cyclohexane, n-hexane, sodium methoxide, sodium hydroxide, potassium iodide, Wijs reagent, starch, and silica Gel 60 No. 7734, tetrahydrofuran were obtained from Merck (Darmstadt, Germany). The reference mix of rapeseed oil (i.e., RM-5 and RM-6 methyl esters) were purchased from Supelco (Dorset, UK). Ultra-pure water was used during the analysis (Purelab Classic UV, Elga Labwater, Lane End, UK). Solid-phase extraction cartridges Oasis Hydrophilic–Lipophilic Balance (HLB), 3cc and Oasis Mixed-mode Cation-eXchange (MCX), 3cc, were supplied by Waters Corp. (Milford, MA, USA). Acetic acid, isopropanol, petroleum ether, diethyl ether, were purchased from Systerm (Shah Alam, Malaysia).

A mixture of calcined potassium bromide (200 g), hydrobromic acid (10 mL), and bromine-saturated water (saturated at 4 °C, 160 mL) and 830 mL distilled water were prepared as a bromination reagent and kept at 4 °C. Acrylamide and ^13^C3-labelled acrylamide were solubilized in distilled water with concentrations 0.2 mg/mL and 4 µg/mL respectively as stock solutions. Two working standard preparations with concentrations of 1 µg/mL and 10 µg/mL, respectively, were prepared with a further dilution of the acrylamide stock solution with water. All stock solutions and working standards were kept at 4 °C.

#### 2.3.2. Oil Oxidative Rancidity

##### Determination of Peroxide Value (PV)

The peroxide value was determined according to the AOCS Official Method Cd 8b–90 [37]. The oil sample was mixed with acetic acid-iso-octane (60:40, *v*/*v*) solution, followed by a solution of saturated potassium iodide. The mixture was then titrated with a standardized sodium thiosulfate solution (0.1 mol/L) until the yellowish iodine color turned pale. A starch solution (5 g/L) was then introduced drop-wise, and the titration was finished when the blue color vanished.

##### Determination of *p*-Anisidine Value (*p*-AV)

The *p*-anisidine value analysis was conducted according to the AOCS Official Method Cd 18–90 [38]. The oil sample was first mixed with iso-octane before an anisidine reagent was incorporated. The anisidine value was obtained at a wavelength of 350 nm using a Lambda 35 Spectrometer (Perkin Elmer, MA, USA).

#### 2.3.3. Determination of Free Fatty Acid (FFA)

The FFA content was performed using a titration method described by the AOCS Official Method [39]. The oil sample was mixed with neutralized iso-propanol with phenolphthalein as the indicator. The mixture was then titrated with sodium hydroxide until a pink color was observed for at least 30 s. The FFA content was reported in percentage. 

#### 2.3.4. Determination of Total Polar Compounds (TPC) and Polar Compound Fractions 

The TPC was determined using the silica column chromatography method described by IUPAC [40] with slight modifications. The oil sample was mixed with PE/DE (87:13 *v*/*v*). The mixture was then put into a 4.5 m glass column, initially packed with Silica Gel 60 No. 7734, containing a PE-DE mixture and sea sand. The mixture was isolated into polar and non-polar fractions. First, the non-polar fraction was initially extracted through the column into a flask for 60 to 70 min containing 150 mL of the PE-DE mixture. The column outlet was then flushed with the PE-DE mixture to ensure complete separation. 

For the polar fraction, the same protocols were applied, but for the second extraction, PE was used as the solvent instead of the PE-DE mixture. A rotary evaporator (Büchi Labortechnik AG, Flawil, Switzerland) was then used to remove the solvent (PE-DE mixture) from the fractions. The residue was flushed with nitrogen to ensure the complete removal of remaining solvents. The amount of polar fractions was divided with the mass of the sample to calculate the polar compounds, which were expressed in percentage.

The polar compounds were further quantified for polymerized triacylglycerols (PTAG), oxidized triacylglycerols (OxTAG), diacylglycerols (DAG), monoacylglycerols (MAG), and free fatty acids (FFA). The analysis was conducted according to IUPAC 2.507 with modifications [41] using HPLC with an Evaporative Light Scattering Detector (Agilent 1260 Infinity, Santa Clara, CA, USA). Tetrahydrofuran (THF) was used to dissolve the polar fraction at a 5 mg/mL concentration and initially infiltrated with 0.45 μm nylon membrane filter and injected into the HPLC-ELSD installed with a total of 3 columns packed using PLgel 500A with a 5 μm particle size (7.5 mm × 300 mm) (Agilent 1260 Infinity, Santa Clara, CA, USA). 

Using THF as mobile phase with a flowrate of 1 mL/min, the columns were programmed at 40 °C whereas the evaporator and nebulizer of the ELSD were fixed at 40 °C and 30 °C, respectively. The flowrate of nitrogen for ELSD was set at 1.7 standard L/min (SLM). The classification of polar compound fractions was done according to molecular size. The analysis was performed for 30 min for one complete run.

#### 2.3.5. Determination of Fatty Acid Composition (FAC)

The FAC was determined using a gas chromatography (GC) (Hewlett-Packard 6890 Series). The GC was set with a BPX70 capillary column (60 m × 0.25 mm, i.d. 0.25 μm film thickness), flame ionization detector (FID), electronic integrator, and data processor (J & W Scientific, Folsom, CA, USA). AOCS Official Method Ce 1i-07 was adapted for fatty acid methyl ester (FAME) preparation [41]. Helium was used as the carrier gas at a flowrate of 0.8 mL/min with a pre-column split ratio of 100:1. The sample injection volume was 1 μL. The FID detector and injector port temperatures were set at 240 °C while the column temperature remained isothermal at an oven temperature of 185 °C. Each FAME was quantified and expressed in percentage with the rapeseed oil references standards. 

#### 2.3.6. Acrylamide Analysis

The acrylamide extraction method followed Sanny et al. [42] with slight modifications. A sub-sample of 2 g of ground sample was weighed in a 50 mL centrifuge tube containing 10 mL of ^13^C3-labelled acrylamide with a concentration of 200 ng/mL as the internal standard. The mixture was shaken using a vertical shaker (RS-1, Jeio Tech Co., Gyeonggi-do, Korea) at medium speed level (ca. 256 pulses/min) for 30 min and centrifuged in a refrigerated centrifuge (3–18 K, Sigma, Gillingham Dorset, UK) at 10956 RCF (g) for 30 min. Oasis HLB and MCX were conditioned with 3 mL of methanol followed by 3 mL of water. Next, 5 mL of the supernatant from the centrifuge was directly infiltrated with an Oasis HLB cartridge joined with an Oasis MCX cartridge to obtain the elute.

A matrix matching calibration curve was prepared by spiking 2 g of blank sample (par-fried nuggets) with a series of acrylamide standards at concentrations of (0, 10, 50, and 250) ng/mL and isotopically labelled acrylamide at 200 ng/mL. About 5 mL of elute or standard was added with the bromination reagent (15 mL) for 2 h at 4 °C. Few drops of 0.7 M sodium thiosulphate solution were added until the yellow color vanished to dissolve the excess bromide. Calcined sodium sulphate anhydrous (4 g) was incorporated into the mixture and stirred using a magnetic stirrer for 20 min. The mixture was transferred to a 50 mL centrifuge tube and then extracted twice with 15 mL of ethyl acetate/hexane (4:1, *v*/*v*) by shaking using a vertical shaker for 1 min. After the phase separation, the upper organic phase was collected and transferred into another 50 mL centrifuge tube containing 4 g calcined sodium sulphate anhydrous and centrifuged at 10,956 RCF (g) for 10 min. The liquid phase was filtered through glass wool, into a 20-mL test tube. The collected fraction was evaporated via vacuum at 40 °C using a dry block heater (Barnstead Lab-Line, Melrose Park, USA). The remnant was liquefied with 450 µL of ethyl acetate followed with 50 µL of triethylamine to convert 2,3-dibromopropionamide to 2-bromopropenamide. The final solution was placed into an amber vial and stored at −18 °C before further analysis using gas chromatograph-mass spectrometry (GC-MS).

Brominated sample extracts and calibration standards were analyzed using a GC (Agilent Technologies 6890 Series) (Agilent Technologies, Palo Alto, CA, USA) coupled to a 5973 Network Mass Selective Detector (MSD) with HP-Innowax capillary column (30 m × 0.25 mm i.d., 0.25 m film thickness) (Agilent Technologies, Palo Alto, CA, USA). The carrier gas was helium with a flow rate of 1.6 mL/min. The run time analysis was 30 min of which the column was held at 65 °C for 1 min, then programmed at 15 °C/min to 170 °C, 5 °C/min to 200 °C, followed by 40 °C/min to 250 °C, and held for 15 min at 250 °C. Injections of 2 µL volume were performed in splitless mode (split-flow 60 mL/min) with a purge activation time of 1.0 min and an injection temperature of 250 °C. Ions targeted were *m*/*z* 70, 149, and 151 for 2-bromopropenamide, and *m*/*z* 110 and 154 for 2-bromo(^13^C3) propenamide. For quantification, the peak area with an *m*/*z* ratio of 149/154 and acrylamide standards was used to form a linear calibration curve. The acrylamide level in the samples was then calculated from the plotted linear calibration curve (*r*^2^ = 0.98). The limit of detection was 0.20 ng/g whereas the recoveries average was 112.56%.

#### 2.3.7. Statistical Analysis

All analyses were run in triplicate. The average value and the standard error were calculated using Microsoft Excel software. Two-way analysis of variance (ANOVA) with two independent variables (i.e., type of oils and frying cycles) and their interaction effect as well as Tukey’s pair-wise comparisons were carried out on PV, *p*-AV, TOTOX, FFA, TPC, and acrylamide using statistical software (Minitab 18; Minitab Inc., State College, PA, USA). However, one-way ANOVA was used to analyze the FAC of fresh vegetable oils. Bivariate correlation analyses were also carried out. The statistical probability was considered significantly different at the *p* ≤ 0.05 level.

## 3. Results and Discussion

### 3.1. Changes in Oxidative Rancidity

Oil quality attributes such as PV, *p*-AV, TOTOX, FFA, and TPC, have been widely used to assess the quality and safety of both fresh and used oils because of the chemical alterations occurred during frying [43]. The PV is commonly used as an indicator to assess the oxidative state of oils. This parameter indicates primary oxidation, but it is unstable because peroxides are easily degraded at high temperature [32]. Meanwhile, *p*-AV shows the secondary oxidation products of hydroperoxides, i.e., unsaturated aldehydes, which are more stable than primary oxidation products [44]. The TOTOX value measures the total oxidation products, including both primary and secondary products [44]. The changes in PV, *p*-AV, and TOTOX values for different vegetable oils across 80 frying cycles are presented in Table 1. 

The data showed that SBO has the highest initial PV at 4.43 meq O_2_ kg^−1^, followed by SFO (3.74 meq O_2_ kg^−1^), PO (2.10 meq O_2_ kg^−1^), and RPO (1.45 meq O_2_ kg^−1^). The PV significantly (*p* ≤ 0.05) increased during early frying cycles and significantly (*p* ≤ 0.05) decreased toward all oils’ end cycles. Ali et al. [45] reported a similar pattern in PV when chicken nuggets were fried in canola oil, in which the values decreased upon reaching the maximum after 24 h frying. The researchers observed the PV increased from 2.84 meq O_2_ kg^−1^ to 25.14 meq O_2_ kg^−1^ then reduced to 6.81 meq O_2_ kg^−1^ during 0 h to 8 h and after 24 h, respectively [45]. Similarly, Enríquez-Fernández et al. [31] used PO to fry chicken nuggets and reported the PV increased to 11.56 meq O_2_ kg^−1^ after 120 frying cycles from the initial value at 2.35 meq O_2_ kg^−1^ and subsequently reduced to 8.55 meq O_2_ kg^−1^ after 200 frying cycles. The observed trend is due to hydroperoxides’ degradation to carbonyl and aldehyde compounds [46]. Besides, Table 1 shows that RPO displayed the highest PV at 7.76 meq O_2_ kg^−1^ after 80th frying, whereas PO showed the highest PV at 10.2 meq O_2_ kg^−1^ after 48th frying. These contrast to SFO that displayed the highest PV at 16.4 meq O2 kg^−1^, earlier, i.e., after 16th frying, and SBO showed the highest PV at 7.16 meq O^2^ kg^−1^ after 32nd frying. The present study showed that PO possess high oxidative stability as repeatedly demonstrated by other researchers [47,48]. 

Moreover, Table 1 shows that SFO has the highest initial *p*-AV at 6.72, followed by RPO (5.41), SBO (4.45), and PO (2.02). The initial TOTOX values in fresh oils ranged between 6.26 and 14.2. Like PV, the *p*-AV and TOTOX value significantly (*p* ≤ 0.05) increased during early frying cycles and significantly (*p* ≤ 0.05) decreased toward the end of the cycles for all oils. Other researchers reported a similar trend in which the *p*-AV increased and then decreased slightly during frying French fries using canola oil for 49 h [49]. The observed phenomena could be due to carbonyl content changes or elevated used temperatures that probably caused the diminishing of these labile components [45,50]. However, Abdulkarim et al. [51] reported an increase in the *p*-AV and TOTOX during frying of potato chips for 30 h and by Lim et al. [22] during frying of French fries for 2 h. Aladedunye and Przybylski [49] explained that the increase in *p*-AV could be related to the initial amounts of dissolved oxygen in the frying oil and potential thermo-oxidative degradation stimulated by its presence. In *p*-AV, the degradation is due to the rapid stage of secondary oxidation being transformed into higher molecular weight compounds [46]. The variation in *p*-AV and TOTOX values obtained is the implication of different food compositions used in the frying study affecting the different oil degradation rates [31].

### 3.2. Change in FFA

Oil acidity in terms of FFA mainly measures the degree of hydrolysis of triglycerides and the decomposition of hydroperoxide at high temperatures in the presence of moisture and air [48]. Table 2 summarizes the changes to FFA for different vegetable oils across 80 frying cycles to produce beef nuggets at 180 °C. The results show that the FFA ranged differently for different oils, namely 0.06% to 0.86% for PO, 0.05% to 0.54% for SBO, 0.10% to 0.56% for SBO, and 0.06% to 0.70% for RPO. Generally, FFA increased significantly (*p* ≤ 0.05) across the frying cycles for all oils. The results obtained are consistent with Enríquez-Fernández et al. [31] who used PO to fry chicken nuggets and reported that FFA increased from 0.10 to 0.45% across 200 frying cycles. In addition, Flores-Alvarez et al. [52] reported that the FFA of oil blend containing PO and high-oleic canola oil increased from 0.08% to 1.1% after 12 days of frying fish nuggets. Similar to PV and *p*-AV, the variation in FFA transient is mainly due to different food compositions used in the frying study, which in turn contribute to a different rate of oil degradation [31]. According to Ahmad Tarmizi [46], the discard point for FFA is influenced by the type of food being fried, in which a typical range of 2% to 2.5% is normally obtained for breaded and coated food such as nuggets, fillets, and chicken parts. Thus, based on the data tabulated in (Table 2), the FFA obtained was below the discard point for all the oils. 

### 3.3. Changes in TPC and Polar Fractions

In principle, TPC refers to the polymers of TAG, oxidized TAG, and hydrolysis products, as diglycerides, monoglycerides, and FFA, including all the main products resulting from TAG oxidation and hydrolysis [43]. As shown in (Table 3), the TPC of all oils significantly (*p* ≤ 0.05) increased across the 80 frying cycles. 

Although no significant difference in TPC for all oils after the 80th frying cycle was observed, the highest TPC was observed for RPO (33.04%), followed by PO (31.68%), SBO (29.61%), and SFO (29.12%). According to Ahmad Tarmizi et al. [53] and Bansal, Zhou, Barlow, Lo, and Neo [30], the higher TPC in PO was due to its base content of DAG. 

Table 3 shows that even in fresh PO and RPO, a higher content of DAG was observed compared to that in SFO and SBO. These data are similar to that of Ahmad Tarmizi, Hishamuddin, and Abd Razak [53], who found that the content of DAG in PO was four times higher compared to other oils. The changes in polar fractionations are summarized in (Table 3) to provide more precise information on the breakdown of the compounds based on polarity differentiation, which reflects the respective oil degradation pathways [54]. It can be observed that the amount of polymerized TAG (PTAG) increased across the 80 frying cycles, with the highest PTAG observed in SBO (16.20%), and the lowest in PO (10.29%). The PTAG is the product of the tertiary oxidation and thermal degradation of frying oil [53]. This finding is consistent with that of a previous study [55], which found that the PTAG of oils increased with increased frying time. A similar trend was observed for all oils in this study, where the oxidized TAG (OxTAG) content increased across the 80 frying cycles. The lowest OxTAG was observed in SFO (9.81%) whereas RPO exhibited the highest amount of OxTAG, which was 13.5%. Aladedunye and Przybylski [55] explained that OxTAG are primary products deposited from primary and secondary oxidation during frying.

### 3.4. Changes in the Fatty Acid Composition

Table 4 shows the FAC of fresh vegetable oils used in the frying experiment. A high content of palmitic acid (C16:0) and oleic acid (C18:1) was observed in PO and RPO. Meanwhile, SFO and SBO mainly comprised of oleic acid (C18:1) and linoleic acid (C18:2). The results are consistent with other researchers [46,53] who reported a similar composition of the above-mentioned fatty acids in oils under investigation. Polyene index was calculated, and it was determined the lowest in PO and the highest in SFO. PO has been repeatedly shown to have higher resistance toward oil oxidation [47,48]. Ahmad Tarmizi [46] stated that oil with a high content of saturated fatty acids and low content of unsaturated fatty acids has higher oxidative stability. 

**Figure 1:**
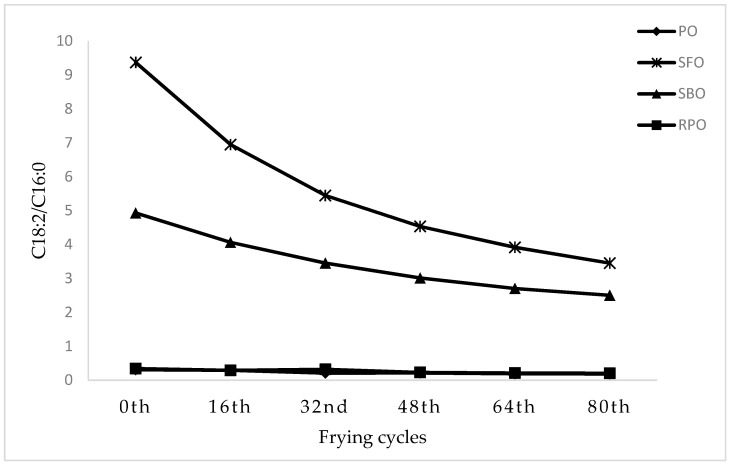
Changes in the ratio of C18:2/C16:0 across 80 frying cycles in PO, palm olein; SFO, sunflower oil; SBO, soy bean oil; RPO, red palm olein.

The changes in FAC during frying are recognized as one of the crucial indicators of oil stability [56]. Figure 1 indicates the changes in the ratio of C18:2/C16:0 across 80 frying cycles for all oils. The C18:2/C16:0 dropped progressively in SFO and SBO while remained almost unchanged in PO and RPO. Serjouie et al. [57] stated that the primary and secondary oxidation of unsaturated fatty acid causes the decrement in linoleic acid (C18:2) content and this fatty acid is claimed to be less stable to oxidation compared to palmitic acid (C16:0). Thus, the ratio of C18:2/C16:0 is recognized as an oxidative indicator in frying oil [53,56,57]. A similar finding was reported by Ahmad Tarmizi, Hishamuddin, and Abd Razak [53] in which they reported the ratio of C18:2/C16:0 of various vegetables oils was reduced as frying time increased.

### 3.5. Changes in Acrylamide Concentration

The changes in acrylamide concentration in beef nuggets across 80 cycles are tabulated in Table 5. 

The result shows that the types of oils significantly (*p* ≤ 0.05) affected acrylamide formation. The mean acrylamide in the nuggets was calculated across the 80 frying cycles to allow the comparison of acrylamide content for the different vegetable oils. Interestingly, the average acrylamide concentration was significantly lower when PO (327 ng/g) was used compared to SFO (521 ng/g), SBO (613 ng/g), and RPO (808 ng/g). PO is the most suited oil for frying among the vegetable oils studied in the present study as the lowest acrylamide concentration was observed when the PO was used while RPO gave the highest acrylamide concentration. The finding is consistent with the data reported by other publications [10,58] who established that the PO gave the lowest acrylamide content. The type of vegetable oil used in the present study varied in term of unsaturation levels, as shown in (Table 4). The relative degree of unsaturation in vegetable oils, from the lowest to the highest, is as follows: PO < RPO < SBO < SFO. Previous researchers who studied the effect of the unsaturation level of vegetable oils on acrylamide did not use SFO but mostly compared the performance of PO to represent less saturated oil, with SBO, to represent more unsaturated oils [22,59]. For example, Daniali, Jinap, Hajeb, Sanny, and Tan [23] reported lower production of acrylamide using less unsaturated cooking oil compared to more unsaturated cooking oil. 

Daniali et al. [60] also found lower acrylamide amounts in PO (1422 ng/g) than SBO (2447 ng/g). They also suggested that since acrolein could be produced as a lipid oxidation product, SBO, which is susceptible to oil oxidation because of its high unsaturation level, produced higher amounts of acrylamide. The finding is, however, contrary to previous studies that reported the type of oil [27] and FAC [28] do not give a significant effect on acrylamide concentration. Additionally, Jin, Wu, and Zhang [8] reported that acrylamide that forms through lipid oxidation might be prevented with the introduction of antioxidants. Since acrylamide is formed at a high processing temperature, it may reduce the effect of heat-sensitive antioxidants [8]. In the present study, the type of vegetable oil used had varying levels of naturally present fat-soluble antioxidants. Sundram et al. [61] reported that the highest total vitamin E content was found in SBO (902.2 µg/mL), followed by PO (547.5 µg/mL) and SFO (547.5 µg/mL). Meanwhile, red palm oil contains 600 µg/mL carotenes and 900 µg/mL total vitamin E [62]. However, the finding contradicts previous findings reported by Kamarudin, Jinap, Sukor, Foo, and Sanny [59], who showed that the lowest acrylamide was formed when “Carotino” red palm oil was used for the deep-fat frying of French fries because of its high level of antioxidants. This result might be due to the different grade of RPO used in the present study, i.e., industrial-grade RPO containing 100% RPO, in contrast to the Carotino red palm oil, i.e., a blend of 20% RPO with canola oil [59]. Thus, it is postulated that the higher amount of vitamin E level in the industrial-grade RPO used in this study reacted as pro-oxidants instead of anti-oxidants per Kamarudin et al. [59]. The higher total vitamin E content, as well as the higher level of unsaturation in soybean oil compared to palm oil as discussed above, might also explain the findings of the present study where higher acrylamide concentration was found in the nuggets when SBO (613 ng/g) was used rather than PO (327 ng/g). The present study measured the FAC but not vitamin E content. Future studies should include antioxidant analysis to confirm this hypothesis. Becalski et al. [63] and Ehling et al. [64] also observed similar behavior when acrylamide content was higher in potato chips fried in unrefined oils, such as virgin olive oil when compared to frying in refined oils. It is anticipated that the presence of unknown compounds in crude or virgin oils promotes the formation of acrylamide in fried products. However, this hypothesis requires further investigation. Further, Becalski, Lau, Lewis, and Seaman [63] as well as Ehling, Hengel, and Shibamoto [64] suggested that the observed high acrylamide content using unrefined oils was due to the loss of oxidized products and/or carbonyl compound in virgin olive oil during the refining process which is consistent with different researchers [8,9,10] who observed that acrylamide content increases when lipid oxidation products and/or carbonyl compound increases.

Table 5 also shows that the acrylamide concentration insignificantly (*p* ≤ 0.05) varied across the 80 frying cycles. The finding is in line with the data published by other researchers [13,27,28,65], who found that frying cycles did not significantly affect acrylamide formation. However, other researchers reported that acrylamide concentration increased with frying cycles [9,26]. They explained that as frying increases oil oxidation over time, it might also increase the level of carbonyl compounds, which are one of the acrylamide precursors [9,26]. Additionally, bivariate correlation analysis was performed, but no significant (*p* ≤ 0.05) correlation (data not shown) was found between oil quality attributes (PV, *p*-AV, TOTOX, FFA, TPC and polar fraction, as well as FAC) and acrylamide formation in nuggets. 

Kuek, Ahmad Tarmizi, Abd Razak, Jinap, Norliza, and Sanny [58] reported a weak significant correlation between lipid oxidation and hydrolysis and the acrylamide formation in French fries. It is likely that because of the weak relationship, the present study did not observe a significant correlation between oil quality attributes and acrylamide. Although it was previously suggested that the level of carbonyl compounds could increase with oil oxidation, it is difficult for these components in this experiment to react with free asparagine in food to produce acrylamide. Zhang, Zhang, Cheng, Wang, and Qian [12] suggested that only a small amount of oil can infiltrate a nugget during frying since most of the oil is left inside the fryer pot when the nugget is taken out from the oil batch. Therefore, less lipid oxidation products (particularly potential acrylamide precursors) could transfer from the oil to the beef nugget and react with free asparagine [12,66].

## 4. Conclusions

This study showed that the type of oil significantly affected acrylamide concentration in beef nuggets, but frying cycle had no effect. PO is the most suited oil for frying among the vegetable oils studied as the lowest acrylamide concentration was observed when the PO was used. Oil quality results showed an initial increase of PV, *p*-AV, and TOTOX and a gradual decrease after one point for all types of oils. FFA and TPC increased across the 80 frying cycles. The C18:2/C16:0 remained almost unchanged in PO and RPO but dropped progressively in SFO and SBO. Bivariate correlation analysis showed no significant (*p* ≤ 0.05) correlation between oil quality attributes and acrylamide concentration. This result is due to the weak relationship between lipid oxidation and hydrolysis on acrylamide, so the present study did not observe a significant correlation between oil qualities attributes and acrylamide content. 

## Figures and Tables

**Table 1:** Values of oxidative rancidity indexes (PV, *p*-AV, and TOTOX) of different vegetable oils across 80 frying cycles.

		Type of Oils			
Parameters	Frying Cycles	PO	SFO	SBO	RPO
PV (meq O_2_/kg)	0th	2.10 ± 0.04 ^Bb^	3.74 ± 0.22 ^Da^	4.43 ± 0.04 ^Ca^	1.45 ± 0.15 ^Bb^
	16th	7.13 ± 0.09 ^Ab^	16.4 ± 0.14 ^Aa^	6.70 ± 0.61 ^ABb^	8.39 ± 0.56 ^Ab^
	32nd	7.39 ± 1.15 ^Aa^	8.87 ± 0.68 ^Ba^	7.16 ± 0.21 ^Aa^	8.14 ± 0.33 ^Aa^
	48th	10.2 ± 0.61 ^Aa^	5.83 ± 0.04 ^Cb^	5.58 ± 0.06 ^BCb^	7.45 ± 0.57 ^Ab^
	64th	7.49 ± 0.15 ^Aa^	5.79 ± 0.09 ^Cb^	6.15 ± 0.17 ^ABb^	7.48 ± 0.16 ^Aa^
	80th	7.99 ± 0.55 ^Aa^	5.51 ± 0.25 ^CDb^	5.60 ± 0.05 ^BCb^	7.76 ± 0.11 ^Aa^
*p*-AV (unit)	0th	2.02 ± 0.05^E c^	6.72 ± 0.23 ^Da^	4.45 ± 0.28 ^Eb^	5.41 ± 0.33 ^Dab^
	16th	58.2 ± 0.47 ^Ab^	63.1 ± 1.63 ^Aab^	64.1 ± 0.61 ^Aa^	41.4 ± 0.21 ^Cc^
	32nd	59.8 ± 0.32 ^Aa^	61.2 ± 1.23 ^Aba^	58.8 ± 0.78 ^Ba^	56.2 ± 1.24 ^Aa^
	48th	49.3 ± 0.12 ^Bc^	60.2 ± 0.91 ^Aba^	53.2 ± 0.57 ^Cb^	51.2 ± 0.27 ^Bbc^
	64th	46.6 ± 0.61 ^Cb^	55.7 ± 1.12 ^BCa^	49.9 ± 0.83 ^Db^	49.0 ± 0.93 ^Bb^
	80th	44.6 ± 0.28 ^Dc^	51.8 ± 0.23 ^Ca^	47.9 ± 0.37 ^Da^	44.5 ± 0.17 ^Cc^
TOTOX (unit)	0th	6.26 ± 0.04 ^Cb^	14.2 ± 0.66 ^Ea^	13.3 ± 0.35 ^Ca^	8.31 ± 0.63 ^Eb^
	16th	72.4 ± 0.64 ^Ab^	95.8 ± 1.92 ^Aa^	77.4 ± 1.81 ^Ab^	58.2 ± 0.93 ^Dc^
	32nd	71.3 ± 0.44 ^Ab^	82.6 ± 1.83 ^Ba^	73.17 ± 1.21 ^Ab^	72.5 ± 0.58 ^Ab^
	48th	69.8 ± 1.09 ^Aab^	71.9 ± 0.97 ^Ba^	64.3 ± 0.45 ^Bb^	65.6 ± 1.41 ^Bb^
	64th	61.5 ± 0.31 ^Bb^	67.3 ± 0.93 ^CDa^	61.9 ± 1.18 ^Bab^	63.9 ± 1.26 ^BCab^
	80th	60.5 ± 1.37 ^Ba^	62.8 ± 0.28 ^Da^	59.26 ± 0.47 ^Ba^	60.1 ± 0.05 ^CDa^

PO, palm olein; SFO, sunflower oil; SBO, soya bean oil; RPO, red palm olein. Mean value ± standard error (*n* = 2). ^A–E^ Values within the same column with different uppercase letters are significantly different (*p* ≤ 0.05) for each parameter. ^a–c^ Values within the same row with different lowercase letters are significantly different (*p* ≤ 0.05) for each parameter.

**Table 2:** Free fatty acid (FFA) percentages of different vegetable oils across 80 frying cycles.

		Type of Oils			
Parameter	Frying Cycles	PO	SFO	SBO	RPO
FFA (%)	0th	0.06 ± 0.001 ^Fb^	0.05 ± 0.001 ^Db^	0.10 ± 0.001 ^Fa^	0.06 ± 0.001 ^Fb^
	16th	0.18 ± 0.001 ^Eab^	0.14 ± 0.01 ^Db^	0.20 ± 0.01 ^Ea^	0.18 ± 0.001 ^Eab^
	32nd	0.32 ± 0.001 ^Da^	0.24 ± 0.03 ^Ca^	0.29 ± 0.01 ^Da^	0.31 ± 0.01 ^Da^
	48th	0.49 ± 0.001 ^Ca^	0.32 ± 0.01 ^Cc^	0.38 ± 0.01 ^Cb^	0.48 ± 0.01 ^Ca^
	64th	0.70 ± 0.001 ^Ba^	0.43 ± 0.01 ^Bb^	0.48 ± 0.02 ^Bb^	0.61 ± 0.01 ^Ba^
	80th	0.86 ± 0.001 ^Aa^	0.54 ± 0.01 ^Ac^	0.56 ± 0.001 ^Ac^	0.70 ± 0.02 ^Ab^

PO, palm olein; SFO, sunflower oil; SBO, soya bean oil; RPO, red palm olein. Mean value ± standard error (*n* = 2). ^A–F^ Values within the same column with different uppercase letters are significantly different (*p* ≤ 0.05). ^a,b^ Values within the same row with different lowercase letters are significantly different (*p* ≤ 0.05).

**Table 3:** Changes in polar fractions of different vegetable oils across 80 frying cycles.

		Type of Oils			
Parameters	Frying Cycles	PO	SFO	SBO	RPO
Polymerized TAG (%)	0th	0.001 ± 0.001 ^Cc^	0.40 ± 0.02 ^Ba^	0.12 ± 0.01 ^Cb^	0.01 ± 0.01 ^Cc^
	16th	2.81 ± 0.70 ^BCc^	7.50 ± 0.10 ^Aa^	5.92 ± 0.30 ^Bb^	2.51 ± 0.10 ^Cc^
	32nd	5.78 ± 0.20 ^ABc^	12.6 ± 0.60 ^Ab^	14.8 ± 0.20 ^Aa^	6.21 ± 0.10 ^Bc^
	48th	8.18 ± 0.30 ^Ab^	15.3 ± 0.50 ^Aa^	14.1 ± 0.86 ^Aa^	10.1 ± 1.52 ^Aab^
	64th	7.29 ± 2.21 ^ABb^	17.6 ± 0.85 ^Aa^	16.3 ± 0.79 ^Aa^	10.5 ± 0.30 ^Aab^
	80th	10.3 ± 0.10 ^Ab^	16.7 ± 0.33 ^Aa^	16.2 ± 0.71 ^Aa^	11.1 ± 0.14 ^Ab^
Oxidized TAG (%)	0th	0.47 ± 0.01 ^Db^	1.84 ± 0.01 ^BCa^	1.81 ± 0.36 ^Ba^	0.42 ± 0.10 ^Db^
	16th	5.00 ± 0.80 ^Cab^	7.66 ± 0.70 ^Ca^	6.59 ± 0.20 ^ABab^	4.22 ± 0.08 ^CDb^
	32nd	7.88 ± 0.90 ^BCa^	8.52 ± 0.30 ^BCa^	6.54 ± 1.60 ^Aba^	8.81 ± 0.96 ^BCa^
	48th	9.72 ± 0.90 ^ABa^	9.69 ± 0.70 ^BCa^	8.63 ± 1.40 ^Aa^	14.3 ± 1.80 ^Aa^
	64th	11.9 ± 0.07 ^Ab^	10.5 ± 0.30 ^ABc^	11.40 ± 0.20 ^Abc^	13.2 ± 0.10 ^ABa^
	80th	12.6 ± 0.50 ^Aa^	9.81 ± 1.70 ^Aa^	10.9 ± 0.40 ^Aa^	13.5 ± 1.20 ^Aa^
DAG (%)	0th	6.84 ± 0.01 ^Aa^	1.55 ± 0.04 ^BCb^	1.00 ± 0.09 ^Bb^	3.47 ± 0.90 ^Bb^
	16th	8.08 ± 0.20 ^Aa^	1.06 ± 0.05 ^Cc^	1.10 ± 0.01 ^Bc^	5.71 ± 0.20 ^ABb^
	32nd	7.45 ± 0.20 ^Aa^	1.41 ± 0.02 ^BCc^	1.64 ± 0.40 ^Ac^	5.94 ± 0.20 ^ABb^
	48th	7.86 ± 0.01 ^Aa^	1.43 ± 0.03 ^BCb^	1.88 ± 0.30 ^Abb^	7.13 ± 0.50 ^Aa^
	64th	9.54 ± 1.30 ^Aa^	2.01 ± 0.02 ^ABb^	2.11 ± 0.09 ^Abb^	7.42 ± 0.50 ^Aa^
	80th	8.70 ± 0.01 ^Aa^	2.47 ± 0.40 ^Ab^	2.45 ± 0.01 ^Ab^	8.25 ± 0.70 ^Aa^
Total (%)	0th	7.33 ± 0.01 ^Eb^	4.05 ± 0.08 ^Bb^	3.09 ± 0.40 ^Db^	3.96 ± 1.01 ^Db^
	16th	15.9 ± 0.70 ^Da^	16.3 ± 0.50 ^BCa^	13.7 ± 0.50 ^Cab^	12.6 ± 0.30 ^Cb^
	32nd	21.2 ±0.90 ^Ca^	22.7 ± 0.90 ^ABa^	23.2 ± 1.80 ^Ba^	21.0 ± 0.80 ^Ba^
	48th	25.8 ±1.20 ^Ba^	26.6 ± 0.20 ^ABa^	24.8 ± 0.20 ^Ba^	31.5 ± 3.60 ^Ba^
	64th	29.2 ± 0.40 ^ABa^	30.2 ±1.10 ^ABa^	29.9 ± 1.10 ^Aa^	31.2 ± 0.90 ^Aa^
	80th	31.7 ± 0.70 ^Aa^	29.1 ± 2.40 ^Aa^	29.6 ± 0.30 ^Aa^	33.0 ± 1.90 ^Aa^

PO, palm olein; SFO, sunflower oil; SBO, soya bean oil; RPO, red palm olein. Mean value ± standard error (*n* = 2). ^A–E^ Values within the same column with different uppercase letters are significantly different (*p* ≤ 0.05) for each parameterr. ^a–c^ Values within the same row with different lowercase letters are significantly different (*p* ≤ 0.05) for each parameter.

**Table 4:** Fatty acid composition (%) of fresh vegetable oils.

	Type of Oils			
Parameters	PO	SFO	SBO	RPO
C14:0	1.07 ± 0.01 ^a^	0.09 ± 0.01 ^c^	0.09 ± 0.001 ^c^	0.98 ± 0.01 ^b^
C16:0	38.3 ± 0.09 ^a^	6.14 ± 0.06 ^d^	10.7 ± 0.001 ^c^	34.9 ± 0.19 ^b^
C18:0	3.93 ± 0.01 ^a^	3.35 ± 0.001 ^a^	4.36 ± 0.001 ^a^	3.76 ± 0.02 ^a^
C18:1	43.7 ± 0.07 ^b^	31.1 ± 0.03 ^c^	23.3 ± 0.001 ^d^	46.2 ±0.25 ^a^
C18:2	11.8 ± 0.36 ^c^	57.5 ± 0.06 ^a^	52.9 ± 0.001 ^b^	12.1 ± 0.07 ^c^
C18:3	0.31 ± 0.001 ^b^	0.30 ± 0.01 ^b^	7.47 ± 0.001 ^a^	0.31 ± 0.001 ^b^
SFA	43.9 ± 0.20 ^a^	10.5 ± 0.07 ^d^	15.9 ± 0.001 ^c^	40.5 ± 0.22 ^b^
MUFA	43.8 ± 0.16 ^b^	31.2 ± 0.03 ^c^	23.4 ± 0.001 ^d^	46.4 ± 0.33 ^a^
PUFA	12.1 ± 0.36 ^c^	57.8 ± 0.05 ^b^	60.3 ± 0.001 ^a^	12.4 ± 0.07 ^c^
Polyene index (PUFA/SFA)	0.28 ^c^	5.52 ^a^	3.78 ^b^	0.31 ^c^

PO, palm olein; SFO, sunflower oil; SBO, soy bean oil; RPO, red palm olein. Mean value ± standard error (*n* = 2). ^a–d^ Values within the same row with different lowercase letters are significantly different (*p* ≤ 0.05) for each parameter.

**Table 5:** Acrylamide concentration across 80 frying cycles.

		Type of Oils			
Parameter	Frying Cycles	PO	SFO	SBO	RPO
**Acrylamide (ng/g)**	1st	252 ± 22.1 ^d^	451 ± 31.9 ^c^	608 ± 31.7 ^b^	827 ± 17.3 ^a^
	16th	229 ± 29.5 ^c^	566 ± 10.6 ^b^	607 ± 27.5 ^b^	853 ± 44.7 ^a^
	32nd	317 ± 11.8 ^c^	554 ± 28.3 ^b^	602 ± 1.35 ^b^	821 ± 58.3 ^a^
	48th	398 ± 47.4 ^b^	466 ± 14.6 ^b^	605 ± 13.5 ^a^	799 ± 15.2 ^a^
	64th	366 ± 5.19 ^c^	508 ± 13.0 ^b^	596 ± 13.8 ^b^	790 ± 24.9 ^a^
	80th	395 ± 37.6 ^b^	579 ± 69.2 ^ab^	659 ± 53.8 ^ab^	757 ± 18.4 ^a^
	Average	327 ± 9.16 ^d^	521 ± 4.89 ^c^	613 ± 5.22 ^b^	808 ± 29.8 ^a^

PO, palm olein; SFO, sunflower oil; SBO, soy bean oil; RPO, red palm olein. Mean value ± standard error (*n* = 2). ^a–d^ Values within the same row with different lowercase letters are significantly different (*p* ≤ 0.05).

## Data Availability

Not applicable.
